# Hormones as “difference makers” in cognitive and socioemotional aging processes

**DOI:** 10.3389/fpsyg.2014.01595

**Published:** 2015-01-22

**Authors:** Natalie C. Ebner, Hayley Kamin, Vanessa Diaz, Ronald A. Cohen, Kai MacDonald

**Affiliations:** ^1^Department of Psychology, University of FloridaGainesville, FL, USA; ^2^Department of Aging and Geriatric Research, University of FloridaGainesville, FL, USA; ^3^Department of Psychiatry, University of CaliforniaSan Diego, San Diego, CA, USA

**Keywords:** hormones, aging, cognitive functioning, socioemotional functioning, cortisol, estrogen, testosterone, oxytocin

## Abstract

Aging is associated with well-recognized alterations in brain function, some of which are reflected in cognitive decline. While less appreciated, there is also considerable evidence of socioemotional changes later in life, some of which are beneficial. In this review, we examine age-related changes and individual differences in four neuroendocrine systems—cortisol, estrogen, testosterone, and oxytocin—as “difference makers” in these processes. This suite of interrelated hormonal systems actively coordinates regulatory processes in brain and behavior throughout development, and their level and function fluctuate during the aging process. Despite these facts, their specific impact in cognitive and socioemotional aging has received relatively limited study. It is known that chronically elevated levels of the stress hormone cortisol exert neurotoxic effects on the aging brain with negative impacts on cognition and socioemotional functioning. In contrast, the sex hormones estrogen and testosterone appear to have neuroprotective effects in cognitive aging, but may decrease prosociality. Higher levels of the neuropeptide oxytocin benefit socioemotional functioning, but little is known about the effects of oxytocin on cognition or about age-related changes in the oxytocin system. In this paper, we will review the role of these hormones in the context of cognitive and socioemotional aging. In particular, we address the aforementioned gap in the literature by: (1) examining both singular actions and interrelations of these four hormonal systems; (2) exploring their correlations and causal relationships with aspects of cognitive and socioemotional aging; and (3) considering multilevel internal and external influences on these hormone systems within the framework of explanatory pluralism. We conclude with a discussion of promising future research directions.

## INTRODUCTION

Advances in research and technology have extended the human lifespan. Consequently, old and very old individuals are a growing segment of society, and the question of how to maintain or augment cognitive and socioemotional functioning in older age has become an issue of great political, societal, and academic interest. This interest has been further spurred by evidence that some individuals fare better than others in their ability to remain cognitively, socially, and affectively engaged as they age (Tucker-Drob et al., 2014). These improved capacities are often associated with reduced morbidity and mortality (Amieva et al., 2010). An increased understanding of the interplay between the myriad factors that contribute to individual differences in aging trajectories has the potential to inform strategies toward amelioration of negative effects and promotion of life quality among older adults.

These considerations frame the current paper. In it, we review extant research that informs the role of four critical hormone systems—cortisol, estrogen, testosterone, and oxytocin—in age-related changes in brain function. In an attempt to advance research in this area, we focus both on singular and interaction effects of these systems in processes relevant to aging. Conceptually, we are mindful of research indicating that the function of each of these hormone systems is influenced by multiple overlapping and often recursive biopsychosocial factors. Among those are an individual’s genes (Tost et al., 2010; Walum et al., 2012), early life experience (Carpenter et al., 2010; MacDonald, 2012), and current relationships (Schneiderman et al., 2012; Zilioli and Watson, 2012). Thus, our general approach to the neurobiology of the aging process is one of empirically based pluralism (Kendler, 2012). In particular, we propose to conceptualize hormones as “difference makers” worth studying in the context of cognitive and socioemotional aging.

## AGE-RELATED CHANGE IN COGNITIVE AND SOCIOEMOTIONAL FUNCTIONING

As people age, they typically experience declines in various cognitive functions (Alexander et al., 2012; Tucker-Drob et al., 2014). Though there is notable heterogeneity in patterns of change in cognitive function both across and within individuals (Albert et al., 1995; Ram et al., 2011; Schmiedek et al., 2013), studies consistently document age-related decreases in processing speed, reasoning ability, and various memory components (Bopp and Verhaeghen, 2005; Willis and Schaie, 2006; Salthouse, 2010). Alongside the broad cognitive aging literature, there is growing evidence of age-related change in socioemotional domains (Blanchard-Fields, 2007; Scheibe and Carstensen, 2010; Ebner and Fischer, 2014). While some of these changes are characterized by decline, other socioemotional functions remain stable or even improve with age. For example, older compared to young adults are worse in interpreting facial cues related to emotion or trust (Isaacowitz et al., 2007; Castle et al., 2012; Ruffman et al., 2012). Older adults also show increased difficulty with memory for social and emotional information such as names (Crook et al., 1993; Verhaeghen and Salthouse, 1997), faces (Bartlett et al., 1989; Grady et al., 1995), and negative emotional pictures or text (Reed et al., 2014). In contrast, the experience of positive affect increases with age (Charles et al., 2001; Teachman, 2006; English and Carstensen, 2014). Also, older adults become better at some aspects of emotion regulation and emotional problem solving (Kunzmann et al., 2005; Blanchard-Fields, 2007; Urry and Gross, 2010; Voelkle et al., 2013) and often show increased wisdom-related knowledge (Staudinger et al., 1992).

Previous studies have discussed possible mechanisms of cognitive and socioemotional age-related change from psychological, contextual, and biological perspectives (Li et al., 2001; Cabeza et al., 2002; Gazzaley et al., 2005; Ruffman et al., 2008; Ebner and Fischer, 2014). However, numerous open questions remain. In this paper, we propose that the level and function of an interrelated suite of hormones—cortisol, estrogen, testosterone, and oxytocin—operating throughout the body and brain have not been sufficiently addressed in their influence on cognition and socioemotional functioning in older adults.

Our focus on these particular hormones was guided by the conceptual consideration that they represent the discrete but overlapping actions of the hypothalamic–pituitary–adrenocortical (HPA) and the hypothalamic–pituitary–gonadal (HPG) axes. To reduce the number of hormones being discussed, we chose to limit this review to examination of a select set of hormones representative of these larger, interconnected systems. In particular, cortisol is released by the adrenal glands as the end product of a coordinated hormonal cascade. Estrogen is released in concert with progesterone by the ovaries and oxytocin and prolactin by the pituitary, with similar implications for behavior. Examination of these particular hormones provides an interesting contrast in that they mediate responses seen as being antithetical (i.e., cortisol and oxytocin) or important for differences between the sexes (i.e., estrogen and testosterone). Another reason to highlight cortisol, estrogen, testosterone, and oxytocin in the present context is that they constitute active regulators of domains important to the aging process (i.e., stress response, emotional support/bonding, biological transition to older age), as discussed below. Thus, as opposed to presenting an exhaustive review of the multitude of factors affecting cognitive and socioemotional development, we chose to describe four hormones representative of these broader psychological and physiological processes to provide a venue for looking at the interactive effects of hormones in aging.

## INTERPLAY OF HORMONES, BRAIN, AND BEHAVIOR IN AGING

One useful way of conceptualizing the interplay between brain processes, hormonal activity, and behavior is to think of the brain as an endocrine organ. Within this model, the brain both regulates the production of hormones (through the hypothalamus and pituitary gland), and is itself a target for steroid and sex hormones that cross the blood–brain barrier and exert effects in the central nervous system and downstream regions (McEwen et al., 1979; Martignoni et al., 1992). As such, hormones play a central role in physiologic processes and initiation of signaling pathways responsible for development, aging, growth, immunity, reproduction, and behavior. In order to fully appreciate the multifaceted factors that impact cognition and socioemotional functioning it is crucial to have a clear understanding of the dynamics of age-related endocrine change.

Level and function of many hormones change with age (Weinert and Timiras, 2003; Conrad and Bimonte-Nelson, 2010) and, as a consequence, produce a number of psychological and physiological alterations. Typical changes are reduced secretion from peripheral glands and modifications in the central mechanisms controlling hormone release (Chahal and Drake, 2007). This includes reduction in inhibitory systems and dampening of circadian rhythms. These age-related changes in the endocrine system are complex and differ for specific hormones due to a variety of influences, some of which are concomitant with aging. Among those influences are sociodemographic (e.g., ethnicity, social status), lifestyle (e.g., level of physical activity, body mass index, smoking initiation or cessation, diet), and psychological factors (e.g., overall health status, perceived stress, supportive relationships, social integration; Seeman and McEwen, 1996; Uchino et al., 1996). For instance, in older adults, both physical and psychological changes brought about by body mass index, smoking, unemployment, and loss of a partner were associated with accelerations in individual declines in testosterone levels (Travison et al., 2007), while psychological factors like self-esteem and perceived stress contributed to individual differences in cortisol (Liu et al., 2014). Age-related hormonal change can also be a result of pathology associated with disease risk or decreased longevity.

Changes in brain and behavior are rarely attributable to the actions of a single hormone. Rather, they reflect aggregate and widespread changes across multiple hormonal systems, which themselves have recursive interactions with each other (Jankowska et al., 2006; Cappola et al., 2008). Therefore, examination of combined effects of multiple hormones that act simultaneously throughout body and brain is necessary. Based on this consideration we have structured our review in the following way. We first introduce the four hormones, and their physiological roles, with a particular eye toward their function in cognitive and socioemotional aging. We then continue with an integrative discussion of interdependent hormone effects. In this context, we also cover the modulatory role of sex on the relationship between hormones and functional levels in aging. The current literature offers a knowledge base for the effects of cortisol, estrogen, and testosterone in aging. However, to date, very little is known about oxytocin’s age-associated effects and the majority of what is known in this arena stems from animal work. For this reason, in discussing the effects of cortisol, estrogen, and testosterone on cognitive and socioemotional functioning, we leverage work from both young and older adults, while our discussion of oxytocin’s effects is based more exclusively on evidence in young adults. Our discussion is aimed toward developing the central proposal of a multidimensional, systemic, complexity-embracing approach for application in future research.

## CORTISOL: AGING, COGNITION, AND SOCIOEMOTIONAL FUNCTIONING

Cortisol is a steroid hormone released by the HPA axis in response to challenging situations. As the primary effector of the biological stress response in humans, it is implicated in a diverse array of physiologic, metabolic, immunologic, and psychological processes directed toward successful coping (Sapolsky et al., 2000; Kassel and Herrlich, 2007). Cortisol receptors are well-represented in limbic structures involved in affective response (i.e., hippocampus, hypothalamus, amygdala) and in regions central to executive function such as the prefrontal cortex and anterior cingulate cortex (Dedovic et al., 2009; Joëls and Baram, 2009). As a result, the effects of cortisol extend beyond the stress and threat response system to impact mood, attention, and memory (Lupien and McEwen, 1997; Sapolsky et al., 2000; de Kloet et al., 2005). Important also for this field of study is research demonstrating the long-term impact of adverse early experiences and their potential to have a canalizing or “programming” effect on stress hormone and inflammation-modulating systems (Glaesmer et al., 2010; Danese and McEwen, 2012; De Bellis and Zisk, 2014; Matthews et al., 2014).

Recent studies of humans showed negative associations between endogenous morning levels of cortisol and cognitive measures of processing speed (Reynolds et al., 2010) and executive function (Venero et al., 2013). In contrast, evidence indicated positive associations between cortisol levels that were acutely elevated by stress or hydrocortisone administration and inhibitory control (Schlosser et al., 2013) as well as spatial learning (Meyer et al., 2013). Regarding cortisol’s effect on memory, the evidence is currently mixed (Schwabe et al., 2012; van Ast et al., 2013). While cortisol (as induced by stressful experience or acute administration) appears to enhance memory consolidation, it more often impairs memory retrieval. However, this association is not universal and can be modified by dispositional and situational factors such as testing context, emotional arousal, or previous experience.

There also is evidence that effects of cortisol on cognition vary in a dose-dependent fashion. In particular, there is evidence of cognitive improvements under conditions of moderate, time-limited cortisol elevation but evidence of cognitive impairments when cortisol concentrations are persistent or excessively high (Lupien et al., 1999; Abercrombie et al., 2003; Hupbach and Fieman, 2012; Schilling et al., 2013; Moriarty et al., 2014). It is possible that increased motivation for learning and improved coordination of brain regions involved in cognitive operations underlie the cognition-enhancing effects of moderate, short-term cortisol release (Buchanan and Lovallo, 2001; Cahill et al., 2003; Richter-Levin, 2004; Henckens et al., 2012). In contrast, adverse effects of persistent and high levels of cortisol may be a result of atrophy of brain structures critical to memory and reasoning (e.g., hippocampus; McEwan, 1995; Landfield et al., 2007).

A smaller, but growing body of work has addressed associations between cortisol level and socioemotional function. Socially challenging and emotionally evocative contexts such as social rejection and feelings of embarrassment and loneliness elicit cortisol release (Cacioppo et al., 2000; Gunnar et al., 2003; Gruenewald et al., 2004; Dickerson et al., 2008). Higher stress-reactive levels of cortisol have been associated with impaired social competence (Alink et al., 2012), greater withdrawal in social situations (Smider et al., 2002), reduced interpersonal trust (Takahashi et al., 2005), and less engagement in prosocial behaviors (Mathewson et al., 2012), but increased engagement in aggression (Murray-Close et al., 2008; Platje et al., 2013).

The impact of cortisol on key affective and cognitive processes and brain structures associated with those processes suggests that cortisol may play an important role in producing some of the cognitive and socioemotional changes observed in aging. This is particularly likely given evidence of age-associated changes in cortisol level and rhythm (Lupien et al., 1999; Ferrari et al., 2001). In particular, cortisol mean levels increase progressively with age (Laughlin and Barrett-Connor, 2000; Nater et al., 2013). In addition, the typical decline in cortisol across the course of the day is attenuated in aging (Yen and Laughlin, 1998; Heaney et al., 2010; but see Ice et al., 2004). At the same time, cortisol stress responses are often higher in older than young adults (Seeman et al., 2001; Kudielka et al., 2004; Neupert et al., 2006). A meta-analysis of 45 studies reported a significantly larger cortisol response to pharmacological and psychosocial challenge among older compared to young participants (Burke et al., 2005). This effect of aging on cortisol response was about three times greater in women than men. Of note, there were neither age nor sex differences in pre-challenge baseline cortisol levels. This is in line with other studies suggesting that baseline levels of cortisol may not differ between young and older adults (Wolf et al., 2002; Kukolja et al., 2008; but see Kudielka et al., 2004; Agrigoroaei et al., 2013). However, while current cross-sectional studies comparing basal levels of cortisol do not suggest differences between young and older adults, longitudinal studies measuring change in baseline cortisol level with age within individuals may offer a better predictor of risk for cognitive impairment.

Increased cortisol in response to stress has been identified as a feature of a well-functioning HPA axis. That is, some degree of cortisol rise is an adaptive response to stressful situations and also appears important to cognitive function. Evidence for this comes from a recent study that showed that low compared to high cortisol response to acute stress in older adults was associated with poorer declarative and working memory performance (Almela et al., 2014). These findings highlight the dynamic complexity of the study of hormonal stress-responses, as both the dynamic reactivity of the system (hyperactive vs. hypoactive responses to challenges) and its intrinsic modulation (shorter vs. prolonged elevations) can impact outcomes (Lupien et al., 2009).

The negative impacts of cortisol among older adults have been proposed to occur, at least in part, as a result of the wear and tear body and brain experience from persistent activation of the biological stress system (McEwan, 2002; Juster et al., 2010). This allostatic load model appears particularly useful in explaining age-related cognitive decline. Chronic overexposure to cortisol damages brain structures and bodily systems. This in turn accelerates the physiological and cognitive aging process (Li et al., 2006; Lupien et al., 2009; Oitzl et al., 2010). This interpretation is supported by evidence that age-related elevations in endogenous cortisol levels were associated with declines in memory performance (Kalmijn et al., 1998; Lupien et al., 1998; Li et al., 2006; Lee et al., 2007; Huang et al., 2009) and executive function (Fonda et al., 2005; Lee et al., 2007). In addition, prolonged cortisol exposure contributed to hippocampal atrophy and cognitive impairments in aging (Lupien et al., 1998; McEwen et al., 1999).

Notably, the direction of the association between cortisol and functioning in aging is not consistent across all studies and a number of modulating psychosocial influences have been identified. One influence to consider is overall health status. Dysregulation of HPA axis activity is common in dementia and progressive cognitive impairment in aging (Csernansky et al., 2006; Lee et al., 2007; Peavy et al., 2009; but see Schrijvers et al., 2011). Alteration in HPA axis activity is also a hallmark of major depression (Holsboer, 2001; Blazer, 2003), although there are mixed results as to the direction of the cortisol-depression relationship (Fiocco et al., 2006; Wrosch et al., 2007; Chuia et al., 2014). Another modulator with high relevance in aging is supportive relationships and social integration (Seeman and McEwen, 1996; Uchino et al., 1996). There is evidence that chronic feelings of loneliness can strain the HPA system and accelerate the aging process (Hawkley and Cacioppo, 2007). Also, cortisol awakening response was higher among lonelier older individuals (Adam et al., 2006) and in those who reported low social status (Wright and Steptoe, 2005). Furthermore, older adults frequently exposed to negative age stereotypes may experience more stress and, as a consequence, a worse aging trajectory (Taylor et al., 2003; Liu et al., 2014; but see Sindi et al., 2012). This may be a result of the body becoming less resilient with age and less able to modulate the increased physiological arousal caused by adverse emotional states (Piazza et al., 2013). Supportive evidence comes from recent studies showing that subliminal exposure of older adults to negative age stereotype primes was associated with greater cardiovascular stress both before and after engagement in cognitive tasks and predicted worse task performance (Levy et al., 2000; Stein et al., 2002; Hess et al., 2003, 2004; O’Brien and Hummert, 2006). In addition, stereotype threat mediated the relation between age and memory recall performance (Chasteen et al., 2005). These findings suggest that negative age stereotypes may act to directly cause stress to older individuals, in that they exacerbate physiological responses when faced with stressors and negatively affect cognitive function.

## ESTROGEN AND TESTOSTERONE: AGING, COGNITION, AND SOCIOEMOTIONAL FUNCTIONING

The role of the sex steroid hormones estrogen and testosterone in sexually dimorphic physiological characteristics is well-known. There is also increasing evidence that sex hormones influence cognitive and socioemotional functioning in aging. When controlling for factors such as disease status, weight, and alcohol consumption, increasing age was reliably associated with declines in estrogen and testosterone in humans (Ferrini and Barrett-Connor, 1998). In addition, age-associated estrogen and testosterone deficiencies were predictive of increased frailty and other forms of physical decline (Cappola et al., 2009; Horstman et al., 2012).

Steroid hormones act as trophic factors in brain development and plasticity (Peper et al., 2011). They are involved in neurite growth, myelination, and the organization of connections by augmenting synaptic growth and promoting the formation of dendritic branches and neural connections. As it pertains to the aging process, there is evidence of estrogen’s long-term neuroprotective effects in hippocampal aging (Ha et al., 2007). Compared to post-menopausal women, young healthy women had smaller ventricles, less cerebrospinal fluid, and more gray matter. However, post-menopausal women on long-term estrogen supplementation showed a pattern better approximating that of young adults. That is, they showed smaller ventricles and greater white matter volume than post-menopausal women who were not on estrogen supplementation. Hormone exposure did not affect gray matter volumes. Furthermore, as reported in a comprehensive review, the ovarian hormones estrogen and progesterone appear to enhance cortical–cortical and cortical–subcortical connections in the human brain (Peper et al., 2011). For instance, estrogen infusion increased connectivity among the hippocampus and the frontal cortex, as well as the amygdala and the prefrontal cortex, all of which are structures associated with cognitive and emotional processes (Ottowitz et al., 2008).

In addition to these structural effects, the amount of circulating ovarian hormones influenced functional abilities in humans, specifically, verbal memory (Peper et al., 2011) and explicit memory recall (Gooren, 2007). Moreover, estrogen administration was found to protect against neurodegeneration only in cognitively intact women for whom degeneration had not yet started (Siegfried, 2007). A beneficial effect of ovarian hormones on cognitive ability was also shown in the context of Alzheimer’s disease (AD), at least for younger women with no cellular damage (Vest and Pike, 2013). However, once the first signs of neurodegeneration were present, supplemental estrogen increased degeneration and accelerated disease progression (Siegfried, 2007). Thus, one potential reason for greater prevalence of AD in women than in men may be the relatively abrupt decrease of ovarian hormones upon menopause.

The relationship between testosterone levels and cognitive functions is mixed. Some studies document an association between decreasing levels of testosterone in old age and cognitive decline in men (Moffat et al., 2002). Other studies suggest that higher levels of testosterone do not contribute to enhanced cognitive ability (Emmelot-Vonk et al., 2008). Also, while some studies (Holland et al., 2011) suggest a neuroprotective effect of testosterone in older men, other studies do not (Gooren, 2007). It is possible that there is an optimal level of testosterone, which, if surpassed, is not beneficial but rather has negative effects on cognition (Muller et al., 2010; Vest and Pike, 2013). Moreover, the neuroprotective effect reported for testosterone may be the result of its conversion to estrogen in the brain (Garcia-Segura et al., 2001). Or it may be due to direct binding of testosterone to sites with a high density of androgen receptors, such as the hippocampus, a brain structure that is crucial for memory formation (Holland et al., 2011). Notably, estrogen administration trials have failed to show therapeutic effects on present symptoms of AD in women (Henderson et al., 2000; Mulnard et al., 2000). In contrast, some success has been noted for testosterone administration in improving spatial memory (Cherrier et al., 2005) and overall life quality (Lu et al., 2006) in male patients.

In addition to their role in cognition, ovarian hormones have been shown to affect socioemotional functioning. For example, differences in levels of sex hormones may underlie the greater emotional expressivity and increased ability to recognize facial expressions in others seen in women compared to men (Hampson et al., 2006; Kret and De Gelder, 2012). In addition, specific periods of endogenous ovarian hormonal variability have been associated with greater negative mood symptomology in women, such as in premenstrual dysphoric disorder (Andréen et al., 2009; Bäckström et al., 2011). Also, women in the premenstrual phase of their cycles (associated with higher levels of estrogen and progesterone) showed increased activation in the anterior-medial orbitofrontal cortex but lower activation in the lateral orbitofrontal cortex to negative stimuli during an emotional go/no-go task compared to women in the post-menstrual phase (Protopopescu et al., 2005). Similarly, brain activity to positive vs. negative pictures was different in regions such as the medial prefrontal cortex and insula in post-menopausal women treated with estrogen compared to women treated with estrogen plus progestin and untreated women (Shafir et al., 2012). In particular, untreated compared to treated women showed greater activity in the medial prefrontal cortex, insula, and entorhinal cortex during naming of positive pictures.

In contrast with estrogen, high levels of testosterone appear to decrease empathy and increase aggression (Montoya et al., 2012). This is reason to think that, under certain conditions, testosterone may negatively affect aspects of socioemotional functioning. For example, levels of bioavailable testosterone have been associated with greater prevalence of depression as seen in hypogonandal men (Gooren, 2007). Notably, some of these effects of testosterone on socioemotional functioning were sex-specific. This may be due to reliably lower testosterone levels in women than men, which may render women more sensitive to variations in testosterone levels (Bancroft, 2009). For example, in a study conducted with young women, testosterone administration reduced cognitive empathy compared to placebo (van Honk et al., 2011). Similarly, while endogenous testosterone showed a positive association with sexual intimacy, it showed a negative association with nurturant intimacy in both men and women (van Anders et al., 2011). For men, there is further evidence of an association between biologically available testosterone and dominance and aggression (Gray et al., 1991), leading to the influential “challenge hypothesis” of testosterone function (Wingfield et al., 1990). More recent research suggests that the association may be less direct in women, such that an aggression-enhancing effect of naturally occurring high testosterone levels was seen only when cortisol levels were also high (Denson et al., 2013). The importance of recognizing such interaction effects among hormones in their role on cognitive and socioemotional functioning is a central premise of this review and will be discussed in more detail below, with reference to sex modulations and effects on aging.

## OXYTOCIN: AGING, COGNITION, AND SOCIOEMOTIONAL FUNCTIONING

Oxytocin is a neuropeptide with both peripheral and central functions (Gimpl and Fahrenholz, 2001). In humans, though it has been traditionally associated with labor and lactation (Pedersen, 1997), oxytocin receptors have also been found in organs unrelated to reproduction (Gimpl and Fahrenholz, 2001). Behaviorally this is reflected in modulatory effects of oxytocin on a wide spectrum of processes related to cognition and socioemotional functioning (Bartz et al., 2011; Meyer-Lindenberg et al., 2011; Feifel et al., 2012; Szeto et al., 2013). These broad effects include a putative role in neurogenesis by which oxytocin administration stimulates adult neurogenesis in the rodent model (Jafarzadeh et al., 2014) even under conditions of stress and elevated glucocorticoids (Leuner et al., 2012).

Throughout this paper, though we emphasize the oxytocin system, we acknowledge the dynamic interplay between the oxytocin and the arginine vasopressin systems (Neumann and Landgraf, 2012). These two neuropeptides share a distant evolutionary ancestor, and differ by only two amino acids. Though their central actions are often contrasted (Legros, 2001; Neumann and Landgraf, 2012), there is also a potential functional overlap between the central effects of these hormones. In particular, oxytocin has an affinity for arginine vasopressin receptor 1A, the vasopressin receptor subtype found most commonly in the brain (Stoop, 2012). Animal experiments, in fact, indicate that arginine vasopressin receptors may play a role in some of oxytocin’s central effects (Schorscher-Petcu et al., 2010; Sala et al., 2011; Mak et al., 2012). That said, the majority of human administration and genetic studies have focused on oxytocin rather than arginine vasopressin.

There is some evidence that administration of oxytocin improves social memory in animals and humans (Striepens et al., 2011). For example, in humans, it enhances face recognition (Savaskan et al., 2008; Rimmele et al., 2009). As a recent meta-analysis suggested, there are specific improvements found in the recognition of happy and fearful faces, and, under certain conditions, angry faces (Shahrestani et al., 2013). In addition, intranasal oxytocin increases facial trustworthiness and attractiveness ratings (Theodoridou et al., 2009), interpersonal trust, and the willingness to take social risks (Kosfeld et al., 2005; Baumgartner et al., 2008). It also has been shown to influence social approach, attachment, and bonding processes (Scheele et al., 2013). In a reciprocal manner, social bonding can affect plasma oxytocin levels in humans (Schneiderman et al., 2012). Additionally, intranasal oxytocin increased positive relative to negative behaviors during a laboratory couple conflict and reduced post-conflict and stress-elicited cortisol levels (Ditzen et al., 2009; Quirin et al., 2011; Cardoso et al., 2013). The latter finding supports interaction effects between oxytocin and cortisol. This potential stress reducing-effect of oxytocin is further informed by evidence that individuals with increased plasma oxytocin healed faster and had a greater number of positive interactions with partners during a 24-h hospital stay (Gouin et al., 2010; see Taylor et al., 2006 for a discussion of oxytocin’s role during relaxation vs. stress; see also Feldman et al., 2011). Differing theories about the role of oxytocin in socioemotional processes include oxytocin’s role in augmenting the activation of social reward neural circuits, in increasing the salience of social stimuli, in reducing social anxiety, and in promoting social approach in both humans and animals (Yoshida et al., 2009; Zink and Meyer-Lindenberg, 2012; De Dreu, 2014; MacDonald and Feifel, 2014).

Of note, however, there have been inconsistencies across studies reported in the literature. In particular, there is evidence that the response to oxytocin administration varies as a result of personal, contextual, and methodological factors such as sex, genes, distribution and density of oxytocin receptors, and dispositional variables such as trait anxiety, social setting, and specific task instructions and measures (see Bartz et al., 2011; Guastella and MacLeod, 2012, for a balanced discussion).

Though it has been conceptualized as a uniquely “social neuropeptide,” a recent theory suggests that oxytocin’s well-documented social effects are part of its larger, more general function, namely the biasing of approach-avoidance motivational processes (of which social motivations are exemplars; Harari-Dahan and Bernstein, 2014). This more general function frames our current understanding of the effects of oxytocin on non-social cognition. On one hand, several studies suggest a potential amnesic effect of oxytocin administration for non-social information (Ferguson et al., 2000; Rimmele et al., 2009; Herzmann et al., 2012). For example, there is evidence of worsened verbal memory performance after single-dose oxytocin administration (Ferrier et al., 1980; Fehm-Wolfsdorf et al., 1984; Bruins et al., 1992; Heinrichs et al., 2004). Moreover, Ansseau et al. (1987) documented significant amnesia in a patient with obsessive compulsive disorder after use of intransasal oxytocin over 4 weeks. However, this latter result needs to be interpreted with caution given the single-case nature of this study. On the other hand, these previous findings contrast recent work in a sample of schizophrenic patients that showed improved performance following intranasal oxytocin administration over a period of 3 weeks on several measures of non-social verbal memory, with effects particularly pronounced for short-term recall (Feifel et al., 2012).

Critical also in the present context—and different from the work on cortisol or estrogen and testosterone—is that current studies on oxytocin’s role in cognitive and socioemotional functioning in humans have almost exclusively been conducted with young adults (but see Barraza et al., 2013; Campbell et al., 2014). As recently summarized (Ebner et al., 2013; Huffmeijer et al., 2013), the existing evidence on oxytocin and aging in animal research is mixed. Some studies find no noticeable effects of aging on the oxytocin system (Wierda et al., 1991; Arletti et al., 1995), while other studies report age-related change (Fliers and Swaab, 1983; Arsenijevic et al., 1995; Melis et al., 1995). Notably, some of the studies reporting comparability of the oxytocin system across older and young subjects refer to peripheral oxytocin levels (Fliers and Swaab, 1983; Zbuzek et al., 1988; Melis et al., 1992) whereas several of the studies documenting age-related change examine central oxytocin levels (Fliers and Swaab, 1983; Melis et al., 1992; Arsenijevic et al., 1995; Parker et al., 2010). Thus, given that oxytocin has two modes of action, locally, as neurotransmitter, and, peripherally, as a hormone (MacDonald and MacDonald, 2010), it is possible that aging may change oxytocin transmission in the central nervous system but not in the neurohypophyseal-pituitary (i.e., peripheral) system (Melis et al., 1999). The relationship between oxytocin levels in these two “spaces” (brain/cerebrospinal fluid vs. peripheral) and their relationship to brain activity is an active area of exploration (Kagerbauer et al., 2013; Striepens et al., 2013; Crockford et al., 2014). Also, the often profound interpersonal and relational changes associated with advanced age (i.e., loss of partner, reduction of social networks) and evidence of the protective effect of social relationships on age-related cognitive outcomes (Ellwardt et al., 2013) suggest a key role of this profoundly social hormone in the aging process (Feldman, 2012).

## MODULATORY ROLE OF SEX ON THE RELATIONSHIP BETWEEN HORMONES AND FUNCTIONAL LEVELS IN AGING

It is already known that sexual dimorphism marks many aspects of the aging process (e.g., differential disease rates in men vs. women). In this review we consider sex as a contributor to individual differences in the relationship between hormones and functional ability in aging. In particular, there is evidence that cortisol, estrogen, testosterone, and oxytocin show different profiles in men and women, especially as they age. Therefore, interactions between these hormones in young adults may differ from the interplay of these hormones in older adults. These sex-specific effects of hormones on cognitive and socioemotional function in aging have not been sufficiently addressed in the current literature.

Sex differences have been an integral part of our discussion of the effects of the sex hormones estrogen and testosterone among older adults. Similarly, there is evidence for sex-specific effects of cortisol. For example, research in humans suggest that older women (Seeman et al., 1997; Comijs et al., 2010) and young men (Wolf et al., 2001; Schoofs et al., 2013) may be most susceptible to cortisol’s effects in cognitive and socioemotional domains. Also, there is evidence that cortisol is associated with declines in hippocampal volume for older men but not older women (Pruessner et al., 2001; Bouix et al., 2005). Kudielka et al. (2004) highlight the complexity of evaluating age by sex effects in their examination of responses to psychosocial stress. In their study, older women showed the highest plasma cortisol stress response compared to all other groups. In contrast, for salivary cortisol, older men showed a higher response compared to older women. Similarly, young men compared to young women showed a greater salivary cortisol response to a battery of cognitive tests, while older men compared to older women showed lesser salivary cortisol in response to those tasks (Seeman et al., 2001).

Higher stress responses in older women than older men may be related to estrogen changes in post-menopausal women. This is supported by evidence of increased HPA axis responses to psychosocial stress after as compared to before menopause (Lindheim et al., 1992; Kudielka et al., 1999). Animal work also suggests an impact of estrogen on HPA axis regulation in the form of a potentiating effect of estrogen treatment on corticosterone levels (Burgess and Handa, 1992; Carey et al., 1995; Weiser and Handa, 2009). However, few experimental studies have been conducted on this topic in humans and with contradictory results. For example, in young men, a 48-h estradiol application resulted in elevated cortisol responsivity (Kirschbaum et al., 1996). In contrast, longer term estradiol treatment in post-menopausal women did not alter psychosocial stress-induced HPA axis responses (Lindheim et al., 1992; Kudielka et al., 1999; but see Del Rio et al., 1998). Work by Sharma et al. (2014) showed an inhibitory effect of estrogen administration in older women compared to a stimulatory effect of testosterone administration in older men on HPA axis activity. In particular, estrogen heightened cortisol negative feedback of the HPA system, thereby providing a signal to suppress further hormone release. These findings are in line with research that estrogen may act directly on the adrenal gland and central HPA targets to alter HPA axis feedback (Figueiredo et al., 2007). They also suggest a possible beneficial role for estrogen treatment in reducing cortisol hyper-responsiveness in post-menopausal women (Veldhuis et al., 2013). However, among post-menopausal women, estradiol treatment predicted increased negative mood and impaired cognition after a psychosocial stressor (Newhouse et al., 2008, 2010; Dumas et al., 2013). Different findings in animals vs. humans, young vs. older adults, and in response physiological vs. psychological indices of stress highlight the need for more research to elucidate the nature of the estrogen–cortisol relationship. Identification of the modulatory dynamics between estrogen and cortisol are likely to inform understanding of the effects of hormones on cognitive and socioemotional aging, especially for older women who experience declining levels of estrogen after menopause.

Regarding sex-specific effects of oxytocin, there is a broad animal literature documenting distinct roles of oxytocin in males and females (see MacDonald, 2012, for references). In contrast, human research examining the role of oxytocin in the context of cognitive and socioemotional functioning particularly via administration studies has been largely conducted in men. This bias is rapidly changing, with recent studies sending a strong signal of sex differences in the dynamics and actions of the human oxytocin system (MacDonald, 2012). In oxytocin administration studies, sex effects have been demonstrated for oxytocin’s impact on emotional empathy responses (Hurlemann et al., 2010), amygdala response to emotional images and faces (Guastella et al., 2009; Domes et al., 2010; Marsh et al., 2010; Rupp et al., 2014), conversational intimacy and eye contact (Liu et al., 2012), risk taking (Patel et al., 2014), emotional and cardiovascular responses to a social stressor (Kubzansky et al., 2012), and kinship and competition recognition (Fischer-Shofty et al., 2013).

These sex-specific differences can be framed in the context of the long evolutionary history of oxytocin, and its role in sexually dimorphic reproductive imperatives and survival strategies (Carter, 2014). Among the sex differences which influence our understanding of oxytocin’s function is the dominant female role in infant nurturance in most mammalian species. In addition, there are sex differences in relational and stress-regulatory strategies. These suggest that females are more prone to “tend and befriend” (Taylor et al., 2000) while males are biased to “compete and defeat” (David and Lyons-Ruth, 2005; Smeets et al., 2009; Van Vugt, 2009; Gabor et al., 2012). Recent reviews and theoretical proposals address the interactions between the steroid sex hormones estrogen and testosterone and the neuropeptide oxytocin with respect to their differential actions in neural networks activity (van Anders et al., 2011; Bos et al., 2012; McCall and Singer, 2012). They offer a more detailed exploration of the more proximal, neurobiological aspects of distal, phenotypic effects.

One such example is the Steroid/Peptide Theory of Social Bonds (S/P Theory) by van Anders et al. (2011). This theory offers an integration of diverse literatures on different hormones into a single heuristic. In particular, it addresses some of the paradoxes that arise when applying the testosterone “challenge hypothesis” to different types of social bonds. According to the S/P Theory, testosterone moderates the social effects of oxytocin (and also arginine vasopressin), thereby facilitating a distinction between sexual intimacy (associated with high oxytocin and high testosterone) and nurturant intimacy (associated with high oxytocin and low testosterone). The S/P theory—and others like it (see Bos et al., 2012, for a similar construal)—offer a useful perspective to the dynamic, social context- and sex-specific role of neuropeptides (e.g., oxytocin) and steroid hormones (testosterone, and by extension estrogen) over the lifespan. For example, the association of high oxytocin and low testosterone with nurturant intimacy (i.e., loving, warm contact with others) has implications for our understanding of hormonal contributions to changes in partnered sexuality across the lifespan and also for the different social bonds unique to the aging process (i.e., grand-parenting).

Taken together, our understanding of the complex interrelationship between sex hormones, cortisol, oxytocin, and cognitive and socioemotional functioning has grown. However, the modulatory role of sex on the relationship between these different hormones and functional domains, particularly as it pertains to the aging process, is still limited and future research is warranted.

## INTEGRATIVE APPROACH ON THE ROLE OF HORMONES IN COGNITIVE AND SOCIOEMOTIONAL AGING: HORMONES AS “DIFFERENCE MAKERS”

As reviewed above, currently known is that chronically high levels of the stress hormone cortisol exert neurotoxic effects on the aging brain with primarily negative impacts on cognition and socioemotional functioning. In contrast, the sex hormones estrogen and testosterone appear to have neuroprotective effects in cognitive aging, but may decrease prosociality under certain conditions and in interaction with other hormones. A relative newcomer in this arena is the neuropeptide oxytocin, which seems to largely benefit socioemotional functioning but has some mixed effects in this domain and has not yet been studied in the contexts of aging and effects on cognition. Importantly, in addition to singular effects, research relating hormones to cognitive and socioemotional functioning in aging reveals complex, interdependent effects across hormones. Several of these interdependent effects have been discussed throughout the paper and, specifically, in the context of the modulatory role of sex on the relationship between hormones and functional levels in aging.

Despite various publications on these diverse topics, we are still at the beginning of understanding both the specific effects and the interactive relationships of hormones in their role in aging. In this review we have taken an integrative perspective. In particular, we have discussed the wide-ranging effects of cortisol, estrogen, testosterone, and oxytocin, as well as their complex interactions. Thus, we have reached beyond the traditional approach of describing specific functions of individual hormones (e.g., primary behavioral responses such as stress response for cortisol, reproduction or aggression for sex hormones, and mother–infant bonding for oxytocin), and toward a more multidimensional, systemic, complexity-embracing approach.

In line with this approach—and with an eye toward future studies—we propose applying Kendler’s (2012) model of empirical pluralism to the study of hormonal influences in aging. Within this approach, we believe that the hormones reviewed above constitute “difference makers,” which are amenable to both correlative study (i.e., studying correlations between hormone function, brain, and behavior) and mechanistic study (i.e., following an interventionalist model of causality; Kendler and Campbell, 2009). When applied to the investigation of the neurobiology of aging, this approach notes that important difference-making factors are distributed across multiple biological levels of analysis (i.e., genetics, molecular and systems neuroscience, neuropsychology), as well as situated within different social, political and cultural contexts (Kendler, 2012). This perspective allows for an adequate representation of the complex recursive interactions and patchy causality of hormonal effects on the two interrelated but separate domains of cognition and socioemotional functioning. In particular, we propose that future study of hormones and aging will benefit from examination of complex individual-environment interactions and consideration of biological (e.g., sex, genetic, neurochemical, neurostructural, functional), psychological (e.g., personality, experience, coping), and higher-order contextual (e.g., relational events, social milieu) factors (Kendler, 2012). Within this model, so-called difference makers are measurable factors which can be experimentally examined and viewed as risk factors on or across each of these domains. Examples of such factors are parenting styles, stressful life events, or societal support. In particular, hormonal effects are known to vary as a function of inherent biological characteristics related to an individual’s sex as well as genetic makeup and are likely modulated by temporal, relational, and social processes associated with aging. These factors interact with psychological processes and higher-order social-contextual factors, including personality traits, previous experiences, coping styles, and cultural settings. And together they modulate physiological responses, epigenetic modifications, and subsequent hormonal and brain changes across the lifespan.

## PROMISING FUTURE DIRECTIONS

We have identified selected topics (summarized in **Box 1**) which we believe have great potential to advance understanding of the effects of hormonal systems on cognitive and socioemotional aging. We propose a research focus toward description of a comprehensive pattern of hormonal dynamics in aging. Such a program would integrate central and peripheral hormone function and strive to enhance knowledge of hormone-specific as well as global age-related change. Moving forward, a thorough description of other hormones, such as progesterone and vasopressin that appear to act in concert with estrogen and oxytocin, in their role on cognition and socioemotional functioning is warranted. This will further clarify the complex relationship between biological, psychological, and social factors that contribute to individual differences in aging trajectories.

**BOX 1 — Suggested avenues for future research.**

**Description of a comprehensive pattern of hormonal change in aging**

What are the age differences in adulthood in central and peripheral levels and actions of cortisol, estrogen, testosterone, and oxytocin? Do these differences follow a general, hormone-specific model, or is there a global pattern of change associated with aging? To what extent do salivary and blood-based hormone levels tap into central vs. peripheral hormone functions in aging? What are the age-related changes associated with type, density, and specific location of hormonal receptors, in addition to levels of the hormone itself, and to what extent do those changes affect functional levels?

**Systematic investigation of functional specificity of effects**

How does the impact of cortisol, estrogen, testosterone, and oxytocin vary across functional domains? Which cognitive and socioemotional functions benefit – and which suffer – from age-related change in different hormonal levels?

**Consideration of interactive effects among hormones**

How do levels of cortisol, estrogen, testosterone, and oxytocin interact to influence age-related changes in cognitive and socioemotional functioning? What are unique and what are joint functions of central and peripheral hormones in aging? Are levels of one hormone contingent on levels of other hormones? Are particular ratios of hormone production in aging associated with certain patterns of function?

**Examination of modulating role of sex**

To what extent are the effects of cortisol, estrogen, testosterone, and oxytocin in aging modulated by sex? For example, are cortisol’s actions in predicting a worse aging trajectory affected by the effects of sex hormones? How might individual differences in the oxytocin system – known to have sex-dependent effects – interact with other hormonal systems?

**Emphasis on individual genetic and epigenetic variation in the endocrine system**

What genetic polymorphisms are involved in regulation of specific hormones (e.g., *NR3C1* and *FKBP5* for cortisol; *ESR1* and *ESR2* for estrogen; *NR3C4* for testosterone; *OXTR* and *CD38* for oxytocin) and how are those genetic variations associated with functional age-related change? What are epigenetic processes (i.e., methylation, acetylation) that mediate the relationship between hormones and behavior? How does aging influence these epigenetic processes?

**Identification of historical, relational, and environmental influences on hormones**

How do historical parameters (e.g., childhood trauma) influence hormonal factors and outcomes in aging? To what extent do age-related changes in relational influences like quality and quantity of relationships (e.g., loss of partner, reduced social networks, increased loneliness) and environmental factors (e.g., home-dwelling vs. institution) affect the endocrine system? Do hormones serve as mediators of some of the known causal effects of these “external” factors on functional levels in aging?

**Identification of hormonal associations in healthy vs. pathological aging**

How do associations between hormones and functional levels vary across healthy vs. pathological aging trajectories? For example, how does the relation between cortisol activity and depression affect cognition in aging?

Our review and reflections, for the most part, focused on central hormone levels, which we acknowledge may not be equivalent to peripheral levels. For cortisol, high correlations are observed between blood and saliva (Gozansky et al., 2005; Restituto et al., 2008; VanBruggen et al., 2011), allowing researchers to assess salivary cortisol as a proxy for both central and peripheral functions (Hellhammer et al., 2009). However, for oxytocin, the correlations between blood and saliva are less well-understood (Feldman et al., 2011). Of note, there is exciting emerging research in humans of a positive correlation between cerebrospinal fluid and plasma oxytocin concentrations. This evidence validates central and peripheral markers of oxytocin and suggests that measurements of peripheral levels of some hormones may well-reflect central levels (Carson et al., 2014). Therefore, moving forward, it will be crucial to thoroughly examine both salivary and blood-based hormone levels in the attempt to determine overlap and dissociation of central and peripheral hormone functions.

Given the multidirectionality of aging across functional domains, a systematic investigation of functional specificity of endocrine effects will be particularly informative. It will be crucial to consider modulatory effects among various hormones as well as biological factors (including sex), to draw a comprehensive picture of the effects of hormones in cognitive and socioemotional aging. In line with a recently proposed model of the effects of oxytocin in aging (AGeNeS-OT model; Ebner et al., 2013), individual genetic (i.e., neuropeptidergic individuality; MacDonald, 2012) and epigenetic variation in endocrine systems (often as the result of early experience; McGowan, 2012; Bohacek et al., 2013) constitute important factors that need increased attention in future research (Di Napoli et al., 2014). Identification of genetic polymorphisms involved in hormone regulation as well as epigenetic modulations active in the aging process and their associations with functional levels will be particularly crucial in this context. Said differently, within this multi-level explanatory framework, systematic examination of historical, relational, and environmental influences on hormones and on functional levels in aging is needed. Also, examination of the dissociation between hormone-brain-behavior relationships in normal vs. pathological aging has the potential to further inform clinical interventions.

One aim of this paper was to raise consciousness about a “hormonal level of explanation” in brain-based aging processes. We are confident that adoption of a view on hormones as difference markers in cognitive and socioemotional aging in the context of a multidimensional, systemic approach will spur new research and help move forward this exciting domain of inquiry.

## Conflict of Interest Statement

The authors declare that the research was conducted in the absence of any commercial or financial relationships that could be construed as a potential conflict of interest.
